# Chairman’s Address

**Published:** 1900-05

**Authors:** J. A. Stucky

**Affiliations:** Lexington, Ky.


### CHAIRMAN'S ADDRESS.*

BY J. A. STUCKY, M.D.,
LEXINGTON, KY.

It gives me greatest pleasure to meet and to greet you at this the first meeting of the Southern Section of our Society in "Old Kentucky"; and inasmuch as this has already been the best year, from a scientific standpoint, since its organization, I predict that this will also be the best meeting since that time. The Society was organized five years ago, and its membership now consists of more than two hundred.

Its object, as stated in the constitution, is, "The promotion of the science and art of medicine, especially in its relation to laryngology, rhinology and otology; the development of its members in original scientific work, the promotion of social intercourse and the collection of material which shall become a part of its archives."

The qualifications for membership are, that "the candidate be a graduate in medicine, engaged in the active practice of laryngology, rhinology, and otology, and one who has, by his skill and scientific ability, proved himself qualified for membership." The geographical divisions into Eastern, Middle, Western, and Southern Sections is made so as to enable its members to attend at least one meeting each year without great loss of time and money, the supreme object being the bringing and welding together the great brotherhood of practitioners of medicine who are giving their best efforts to advancing and perfecting the science and art of our specialty. Our progress has been a steady advance all along the line. In some instances rapid strides have been made. We live now in an age of investigation, not theorization. That which cannot be practically demonstrated after thorough investigation is quickly disregarded. Medical science to-day is nearer a fixed one than ever before. We no longer rely upon verbal symptoms—not upon what the patient tells us, but what we see and hear—in order to make a diagnosis. With improved methods of illumination we lit-

*Read before the Southern Section of the American Laryngological, Rhinological, and Otological Society, at Louisville Ky., March 30, 1900.

erally explore almost every cavity and crevice connected with the throat, nose and ear. In each of these divisions, during the year past, most satisfactory work has been done, perhaps the greatest progress having been made in otology. It is now generally accepted that in the majority of diseases of the ear, nose and throat, surgical treatment gives the best results. With the discovery of cocain muriate as a local anesthetic, we thought our cup about filled; but now that to this is added the suprarenal extract, we are enabled to perform bloodless as well as painless surgery. No longer in the majority of operations done on the mucous membranes of these organs is the operator embarrassed and the patient annoyed and made faint by the bleeding. Treatment by frequent applications of powders, ointments, douches or sprays, by patient or physician, is now known to be of questionable utility. That each has a place, and an important one, none will deny; but that their frequent and indiscriminate use is productive of injury "goes without saying." The atomizing fiend is rapidly falling into line with the hypodermic fiend; and too great care cannot be exercised in prescribing those drugs which, used locally, so speedily relieve, exhilarate and intoxicate, lest the drug eventually master the patient instead of his disease. Such cases are becoming alarmingly frequent.

We stand upon the threshold of a new century, looking back with pride upon what has been done and anticipating with enthusiasm the possibilities of the future. It is not so much the new that we seek, but the true; not the temporary, but the lasting results.

To the pathologist and bacteriologist we are lasting debtors. In pathology, perhaps, the greatest advance has been made, and we may safely say the advance has just begun. Bacteriology has also accomplished much to aid us in diagnosis and treatment. Its possibilities are just beginning to be appreciated, but they will be fully revealed by the diligent work of the student. I shall not make even a brief report of progress made during the year in the departments of our specialty. With these you are familiar from the excellent reports in our journals, both at home and abroad. The laryngologist has removed the larynx, and the patient not only lives but talks. The otologist has removed the ear-drum and ossicles, and chiseled away large parts of the skull and opened the brain, and the patient not only lives but hears. The rhinologist has rimmed out the nose until it is as smooth as a gun-barrel, and the patient not only smells but snorts. What next?

These great achievements and advances have enabled us to prolong and make useful the life doomed to an early death, and to make tolerable the previously intolerable one.

Our specialty, like others, is the result of the evolution which has given a better classification of diseases and nomenclature. Obscure conditions of disease have been discovered and elucidated. Treatment has been simplified and new methods and measures introduced. All steps of progress made by the medical practitioner are so plainly marked that all may know how and what we are doing. There is no "trust" or "monopoly" formed in order to keep the knowledge we gain from the public and be enriched thereby. Instead, the knowledge is freely given out in current literature and text-books, in lectures and clinics. There can be nothing narrow or selfish in the true ethical and loyal practitioner of medicine. He makes himself the servant of his profession.

The general practitioner and specialist are under equal obligations to each other, and, so far as the laity is concerned, one is bound by the same ethical rules as the other. The question of division of fees between the practitioner and specialist, recently so freely discussed in medical journals, is mentioned only to condemn, as both question and division is unethical and unjust.

Our growth in the South has been a gradual but healthy one. An inherited conservatism akin to timidity, a lack of clinical material as compared with the East and West, has had much to do with the slowness of this growth; but it has grown, and now occupies a position in the medical world of which we are justly proud. Our best men are equal to the best men in any section of our land, but many of them are scarcely known outside of the borders of their own States. Within the last three years I have visited several of the leading medical centers of the South, and have been surprised at the superiority of the work done, as well as deeply gratified at the large amount of valuable clinical suggestions received from men of whom I had never heard until meeting them at a convention like this. Through the Southern Section of our national society we seek not only to know and learn of your work from you, but also desire to introduce you to the medical world. The East, West, and North need some of our conservatism, and we need some of their dash and push. As members of the medical profession we have no right to hide our lights under a bushel by failing to keep in personal and written contact with our brethren. There is danger of lethargy and conservatism leading us to underestimate ourselves and the honor and dignity of the work we do.

We should each stand for more than one to relieve pain and guide disease of the body to a healthful or a peaceful termination. Let us aim to be "all-round men," for we live in an age of overworked bodies, overtasked brains, and jaded hearts, and there is need for scientific and tactful administration of therapeutical remedies which, in a sense, are neither of the organic or inorganic kingdoms, but human. Let us put into our armamentarium more of the moral, sympathetic and tenderly human compassion; more of the life and deeds of the "physician of the old school," Dr. Wm. MacClure, immortalized in "Beside the Bonnie Brier Bush," by Ian Maclaren. While in our daily work we are medically sifting and testing, separating and garnering the "wheat from the chaff," let us also aim at the ideal life. Idealism may not appeal to the practical, but is not our daily routine eminently if not painfully so? Most of us are obliged to work, to save. Upon our labor depends the food, raiment and comforts of our homes. But ignorance and deception and false ideas of life and its purposes surround us. Worldly applause, the desire to possess fame and wealth at any price, even though acquired by means unworthy, the constant ceaseless endeavor to outstrip one's fellow-men, may appeal to those who are filled with a narrow, selfish ambition. But is such a struggle to be imitated or even admired? Is the reputation thus acquired ever enduring? The men whose fame is perennial, whose deeds are praised and glorified, are those whose memories awaken what is best within us with a thrill of enthusiasm. Under these impulses we press on to make for ourselves a like career.

Let me, in conclusion, repeat some of the admonitions of Dr.

Urban Pritchard, recently given in his address to the International Otological Congress:

"Let us regard ourselves as assembled in common brotherhood marching under the triple banner of Laryngology, Rhinology, and Otology, forming part of that army of science which is engaged in overthrowing the foes of humanity—those foes which have Ignorance, Vice and Prejudice for their leaders."

The real value of these gatherings is not to be measured merely by papers and discussions. This is one of the uses, it is true, for interchange of ideas is always good; still, the chief value of this meeting, together with others who are all interested in one common subject, is the kindling of an enthusiasm which should serve to stimulate older and younger members alike to renewed effort in the paths of research and of practical treatment; and I most heartily wish that this meeting of the Southern Section will be successful in all these directions.

